# Stomach flushing technique applied to quantify microplastics in Crocodilians

**DOI:** 10.1016/j.mex.2019.11.013

**Published:** 2019-11-18

**Authors:** Mauricio Gonzalez-Jauregui, Merle Borges-Ramirez, José António L. Barão-Nóbrega, Andrea Escamilla, Ricardo Dzul-Caamal, Jaime Rendón-von Osten

**Affiliations:** a Instituto de Ecología, Pesquerías y Oceanografía del Golfo de México, Universidad Autónoma de Campeche, Mexico; b School of Environment and Life Sciences, University of Salford, UK; c Operation Wallacea, Wallace House, Spilsby, Lincolnshire, UK

**Keywords:** Stomach flushing method to evaluate microplastics, Stomach content, Digestive transit time, Recovery efficiency, Crocodilians, Microplastics exposure, FTIR

## Abstract

The impact of microplastics on wildlife is a recent problem for which methods to evaluate exposure still need development. Being able to identify and quantify microplastics (particles < 5 mm) in the gastric contents of live crocodiles allows us to evaluate exposure, at both individual and population level, and also its contribution as transporter of other contaminants. The method was validated to determine and quantify microplastics in crocodile stomach contents recovered during an experiment where a known amount of this contaminant was given to crocodiles via oral administration. Through stomach flushing we were able to recover more than 80 % of the total volume of microplastic administrated to each crocodile. In summary, the method used during the experiment consists of 1) immobilization of the crocodile; 2) extraction of microplastics from stomach contents obtained through stomach flushing; 3) separation, identification and quantification of recovered microplastic fragments using microscopy and FTIR.

• *Low cost method that uses a small number of materials, does not take long to produce results and can easily be performed in the field or the laboratory.*
• *Effective in extracting stomach contents (95 %).*
• *High (>80 %) and good (>60 %) recovery efficiencies within two and four days after ingestion of microplastics by crocodiles*

## Graphical abstract

**Figure:**
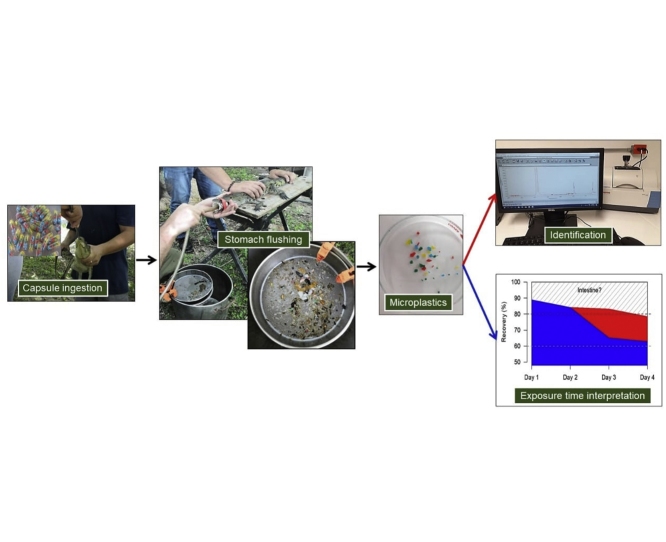

**Specification Table**

**Table:** 

Subject Area:	Environmental Science
More specific subject area:	Pollution and effects in wildlife
Method name:	Stomach flushing method to evaluate microplastics
Name and reference of original method:	Taylor, J. A., G. J. W. Webb, and W. E. Magnusson. 1977. Methods of obtaining stomach contents from Crocodilians (Reptilia, Crocodilidae). Journal of Herpetology 12:415–417. Fitzgerald, L. A. 1989. An evaluation of stomach flushing techniques for crocodilians. Journal of Herpetology 23:170–172.
Resource availability:	Materials: Rope Stainless steel cable snares attached to an aluminium pole Measuring tape Spring scale Insulating tape (vinyl, red) PVC pipe (according to crocodile size) Silicone hose (according to crocodile size) Copper spoon (according to crocodile size) Stainless steel tweezers (precision and 30 cm) Plastic Bucket 20 litre Dissecting forceps (No. 5 and 127 mm) Aluminium tray Reagents: Deionized water Mineral oil Equipment: Dissecting microscope Nicolet iS50 Fourier - Transform Infrared (FTIR) Spectrometer Thermo-Fisher Scientific OMNIC Software for FTIR Spectroscopy and libraries

## Method details

### Protocol background

Worldwide, approximately 320 million tons of plastics are produced per year [1] and many of these end up in aquatic ecosystems as a result of anthropic activities. Most plastics are fragmented into smaller particles known as microplastics (length < 5 m) [2] which tend to deposit in sediments through physical, chemical and biological processes.

Due to their small dimensions, microplastics can be transported across great distances from their original deposition site with relative ease, which in turn facilitates the distribution through the environment of persistent organic pollutants (POPs) and compounds such as hydrocarbons, pesticides, phthalates, among others [3]. These plastics and associated pollutants can be ingested by live organisms that might end up suffering from poisoning effects due to release, filtration and adsorption of these chemical substances [4,5]. After ingestion, plastics can be re-deposited in the sediment by feces or can be transferred along the trophic path to other organisms. Apex predators such as crocodiles, can ingest large quantities of microplastics during their lifetime, either by accidentally consuming plastic parts or by ingestion of microplastic particles accumulated inside their prey. In the past, stomach flushing techniques have been used to obtain stomach contents for dietary studies in crocodilian species [6,7]. These methods of obtaining stomach contents also seem suitable to recover, identify and quantify microplastic particles consumed by crocodilians.

### Material and equipment

Animal Management and Stomach Flushing

• Rope
• Stainless steel cable snares attached to an aluminium pole
• Measuring tape
• Spring scale
• Insulating tape (vinyl, red)
• PVC pipe (according to crocodile size)
• Silicone Hose (according to crocodile size)
• Copper spoon (according to crocodile size)
• 10 L bucket
• Water (purified and de-ionized)
• Mineral oil

Stomach content separation

• Glass vials
• Petri dish
• Deionized water
• Stainless Steel Test Sieve (4.75 mm, 2.36 mm, 1 mm)
• 10 L bucket
• Stainless steel tweezers (precision and 30 cm)
• Aluminium tray

Microplastic extraction, identification and quantification.

• Stainless steel dissecting forceps No. 5
• Petri dish
• Deionized water
• Dissecting microscope
• Nicolet iS50 Fourier Transform Infrared (FTIR) Spectrometer Thermo-Fisher Scientific

### Procedure

Stomach flushing

**Fig. 1:**
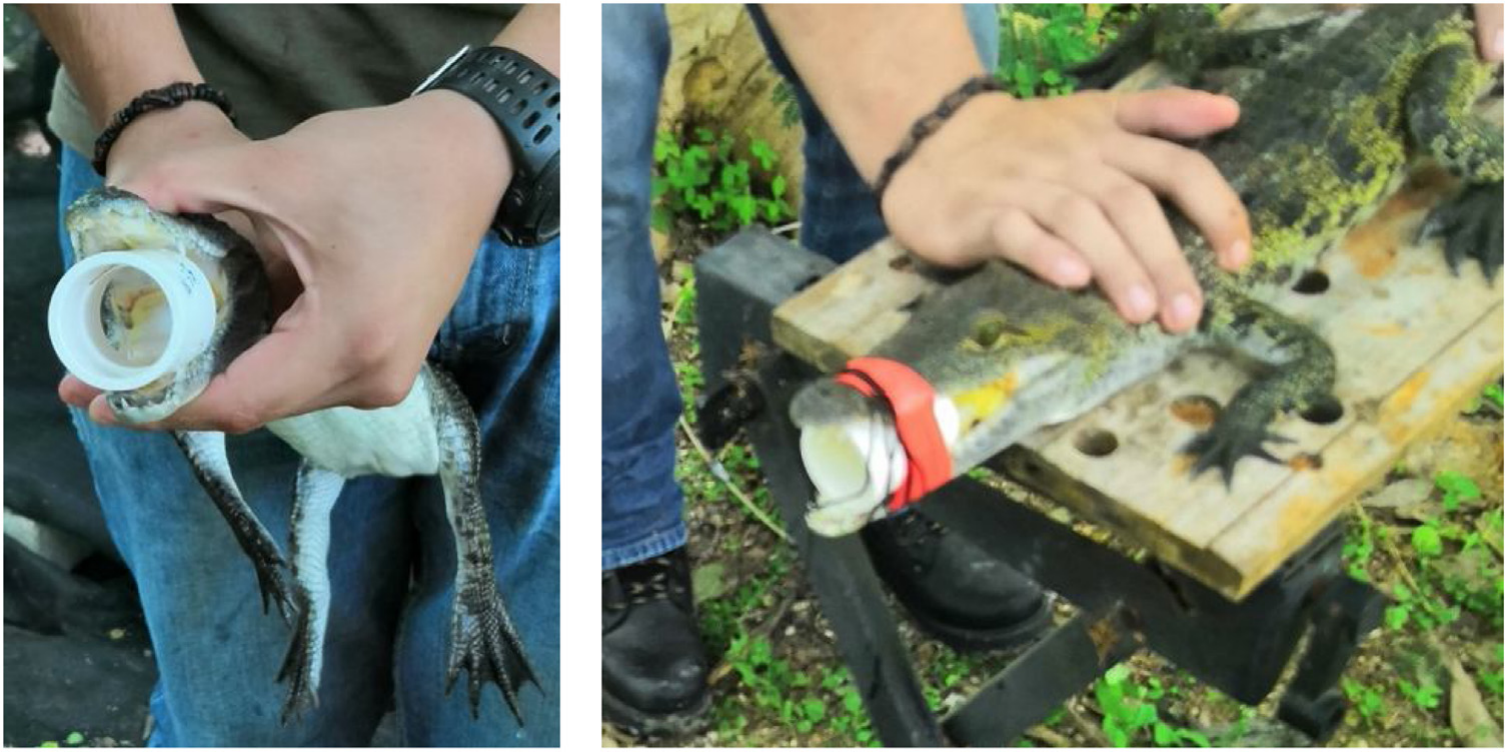
Insertion and fixation of PVC pipe inside the crocodile’s mouth.
• The head of the crocodile was raised at a 30° angle from the body’s longitudinal axis and a copper spoon lubricated with mineral oil was introduced through the PVC pipe and the palatal valve all the way into the stomach (using smooth movements of rotation). The purpose of this step is to assist in the removal of bigger items when the stomach is pumped with water. The choice of which spoon length to use was determined according to each crocodile’s size (Fig. 2).

• Depending on size, crocodiles were either captured by hand or with stainless steel snares attached to an aluminium pole.
• Once the crocodile was captured and securely immobilized, its jaws were manually opened so that a short PVC pipe could be inserted and fixed with vinyl insulation tape (red to be easily seen). Size of PVC pipe was chosen according to crocodile size (Fig. 1).

**Fig. 2:**
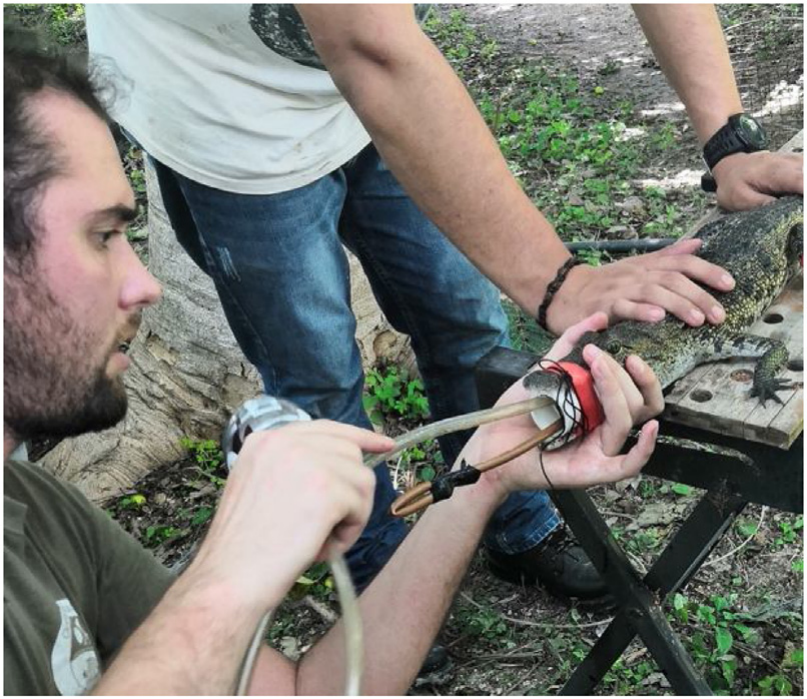
Insertion of the copper spoon and silicone hose (both lubricated with mineral oil) into the digestive tract of the crocodile.
• Once the spoon reached the lower end of the stomach (usually right before the hind limbs, which can be felt by lateral palpation once the spoon is inserted) a silicone hose also lubricated with mineral oil was introduced using the same method. The diameter of the hose should be consistent with the size of the crocodile. When the inserted end reached the stomach, the opposing end was then connected to a funnel through which deionized water was introduced into the crocodile’s stomach (Fig. 3).

**Fig. 3:**
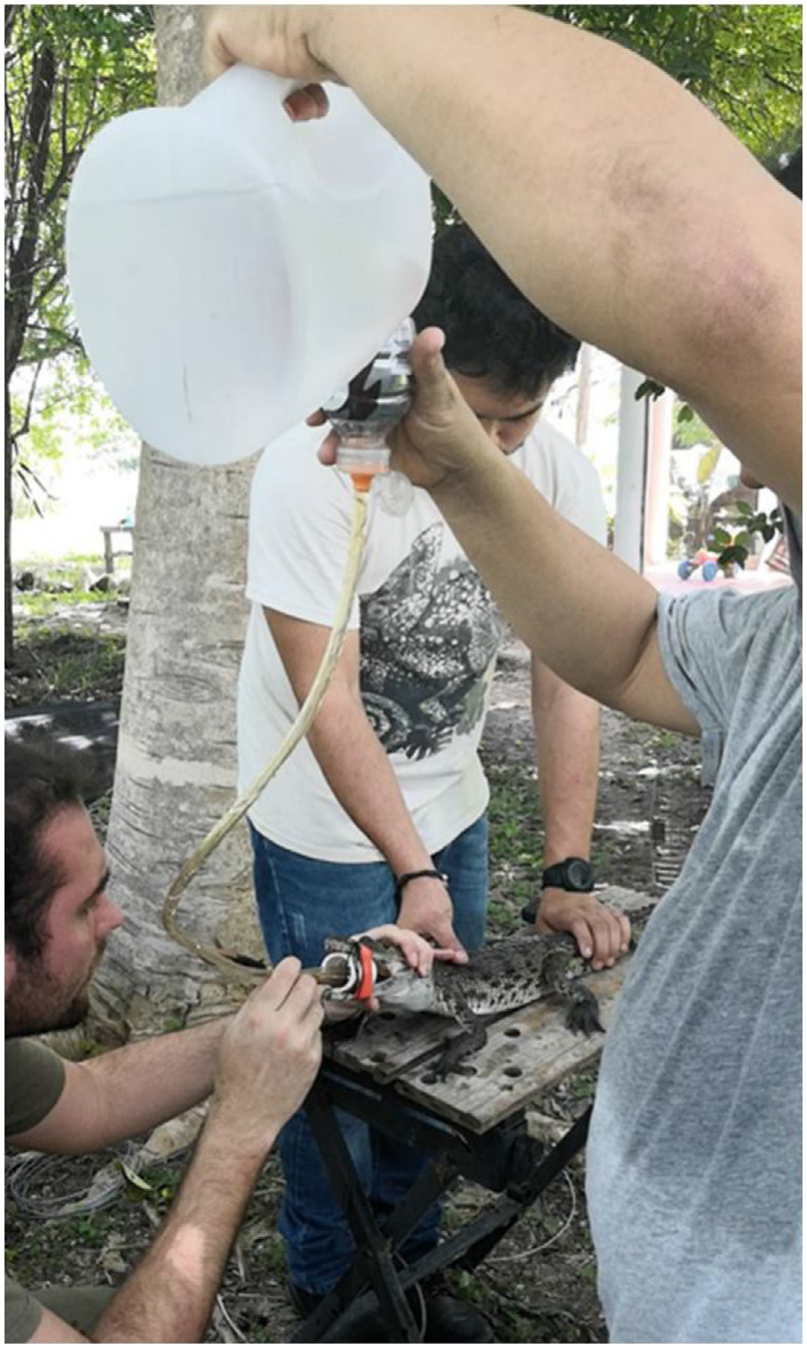
Crocodile stomach filled with purified water.
• Once the stomach was full of water, the hose was quickly removed and the crocodile was tilted at a 45° angle facing a collection bucket with a stainless-steel sieve on top. The metallic spoon was used together with manual stomach massages to assist the removal of stomach contents (Fig. 4).

**Fig. 4:**
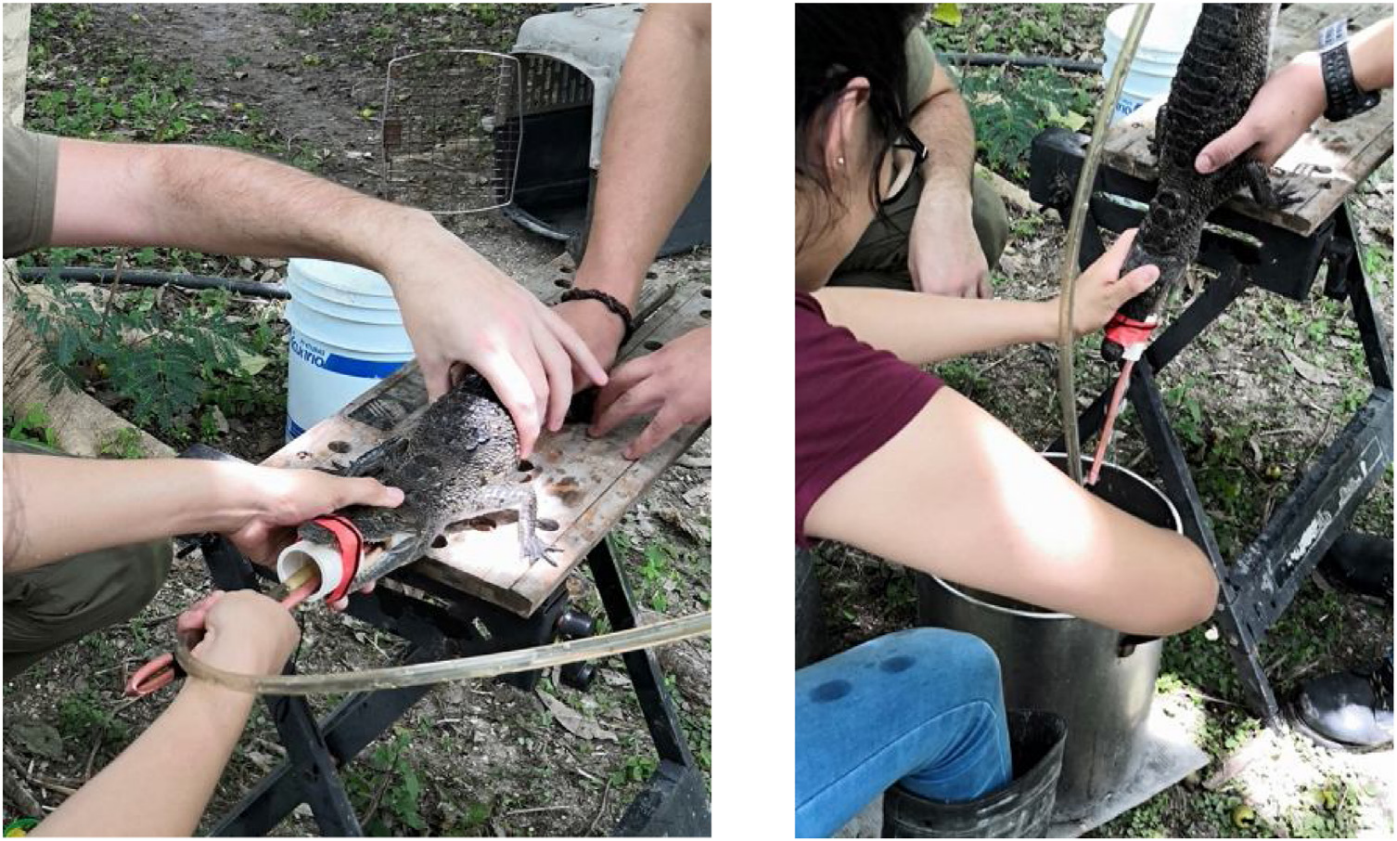
Extraction of stomach contents with the assistance of the metallic spoon and lateral stomach massaging.
• This procedure was repeated four times using purified water or until the expelled contents consisted only of water.
• After the stomach flushing procedures all crocodiles were identified, sexed by visual inspection of the cloaca, measured (snout vent-length and total lengths) and weighted.

Separation of microplastics from stomach contents

• Stomach contents collected inside the bucket were passed through a series of sieves (4.75, 2.36, and 1 mm). All material retained in each sieve was collected, properly labelled and stored in refrigeration. The remaining liquid was properly disposed of.
• In the laboratory, the collected contents were transferred to a petri dish and checked under a microscope (40x) and classified into three groups: i) recognizable fragments of prey; ii) gastroliths and other recognizable materials of natural origin; and iii) possible microplastic fragments
• Fragments of recovered microplastics were collected and labelled.

Microplastic extraction, identification and quantification.

• Possible microplastic fragments were transferred back to petri dishes and each fragment was separated under a dissecting microscope (10×) or an illuminated magnifying glass (4×).
• All suspected microplastic fragments were analysed in a Nicolet iS5 (Thermo) FTIR spectrophotometer in order to identify their composition. Microplastics were identified by OMNIC Software for FTIR Spectroscopy and its libraries.

### Validation

In order to test the efficiency of stomach flushing as a method for quantify microplastics, we developed an experiment using 20 juvenile female individuals of Crocodylus moreletii. Mean total length of crocodiles was 84.12 (±0.52) cm, while snout-vent length was 41.97 (±0.25) cm and weight 1.87 (±0.04) kg. Full morphometric details of all crocodiles used in this experiment can be consulted in the supplementary information.

In order to determine how long it takes for microplastics to pass through the stomach of a crocodile after ingestion, our experiment was carried out over a period of four days. Crocodiles were randomized into four groups of five and each group was put inside a circular tank of about 1 m^3^ filled with water to approximately 10 % of their capacity.

**Fig. 5:**
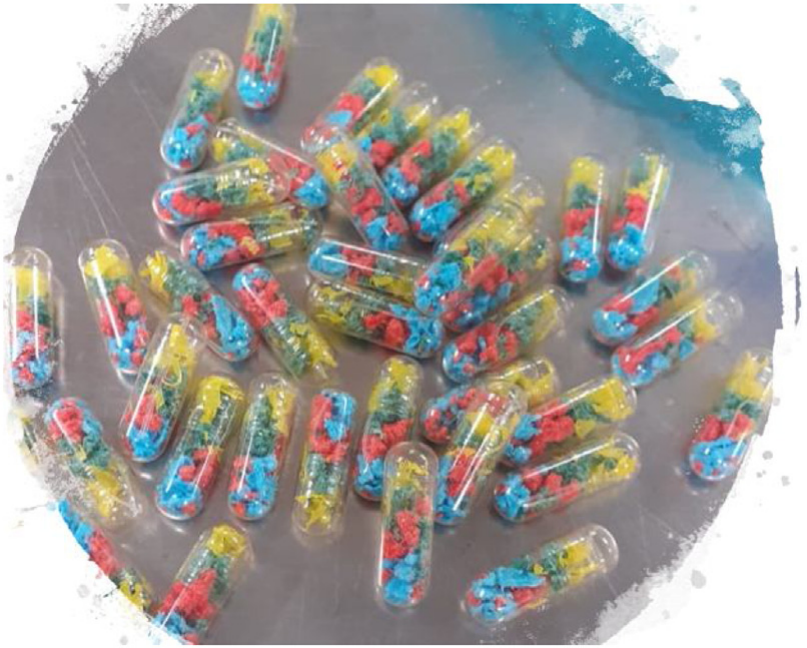
Microplastic capsules ingested by crocodiles during this experiment.

Twenty edible capsules were prepared with 42 microplastic fragments each (Fig. 5), and one capsule was fed to each crocodile at the start of the experiment. In order to easily identify microplastics, materials of different colours were used so that they would create strong contrast against other items in the stomach contents. Size, colour and composition of plastic fragments inserted into each capsule are detailed in Table 1.

**Table 1:** Source materials, composition, colour and size of plastic fragments inserted into in each capsule.

Source Material (Colour)	Composition	Particles per capsule (mm)	
		Size 1 (<4.75, ≥2.36)	Size 2 (≥1, <2.36)
Red (Soda bottle lid)	Polyethylene	5	5
Green (Soda bottle lid)	Polyethylene	5	5
Blue (Soda bottle lid)	Polyethylene	5	5
Yellow (bag)	Polyethylene low density	5	5
Fishing line	Polyamide 6 + Polyamide 6.6	2 (20 x 0.3 mm)	

One capsule was introduced into the esophagus of each crocodile, just behind the palatal valve, using a 30 cm tweezer. After this, pieces of fish fillet were offered and crocodiles were allowed to eat at will.

Twenty-four hours (day 1) after administering the microplastic capsules, stomach contents of all five crocodiles inside the first container were obtained through the stomach flushing technique described above. Remaining water and all floating and deposited particles inside the tank were collected and visually inspected for any excreted plastic fragments. Forty-eight (day 2), seventy-two (day 3) and ninety-six (day 4) hours after ingesting the capsules, crocodiles inside the second, third and fourth tanks, respectively, went through the same procedure as the ones inside the first tank.

Because temperature is important in the metabolism of reptiles, this variable was monitored inside each crocodile tank using HOBO data loggers programmed to take a reading every hour. Mean water temperature inside the tanks was 28.17 °C (24.83–31.06 °C). Complete temperature readings for each tank can be consulted in the supplementary information section.

### Plastic fragments recovered in stomach contents and excretions were classified by colour and analysed through FTIR spectrometry

Overall, we were able to recover 75.3 % of all plastic fragments used in the experiment (71.0 % through stomach flushing and 3.4 % through excretions). The remaining 24.7 % we assumed to be still in transit inside the crocodiles’ intestines at the end of the experiment (day 4).

**Fig. 6:**
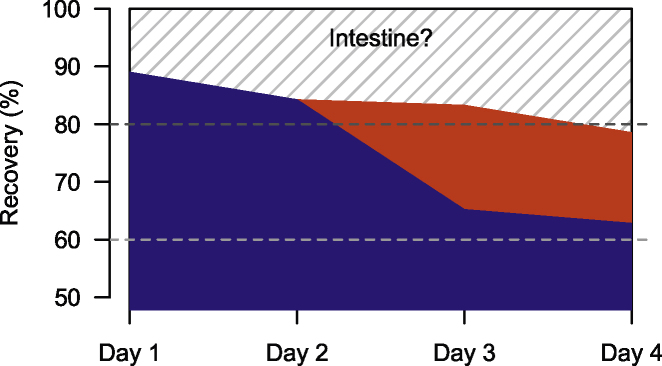
Recovery percentages of plastic fragments ingested by crocodiles. Blue area represents recovery through crocodile stomach flushes and red area through excretions inside the tanks. The shaded area represents the remaining microplastic volume we assume was still in transit inside the intestines at the end of the experiment.

Through stomach flushing we were able to recover a total of 89.0 % of ingested microplastics on the first day, 84.2 % on second day, 65,2 % on third day, and 62.8 % on fourth day (Fig. 6).

Number of recovered plastic fragments in each tank was tested using Kruskal-Wallis non-parametric test, and significant differences were observed between days (Kruskal-Wallis Test Statistic = 24,42 = 16.089, P < 0.01). Mann-Whitney post-hoc pairwise test indicated that recovery was not significantly different between the first and second days after capsule ingestion. However, significant difference was observed between the third and fourth days (Table 2).

**Table 2:** Mann-Whitney post-hoc test probability (p) values of significant difference in plastic recovery rates through stomach flushing between different days.

	Day 1	Day 2	Day 3
**Day 2**	0.78		
**Day 3**	0.02*	0.10	
**Day 4**	0.00*	0.02*	0.78

* *p* < 0.05

These results validate the use of this method for microplastic exposure assessments and monitoring in crocodilians. Microplastic fragments that are recovered from crocodiles and quantified through the described method will indicate recent exposure to this contaminant (likely less than three days).

### Suitability

**Fig. 7:**
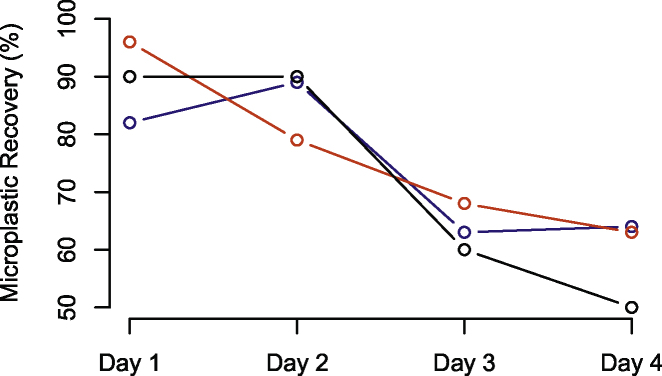
Recovery percentages in relation to ingested fragment size. Blue = Size 1 (between 2.36 and 4.75 mm); Red = Size 2 (between 2.36 and 1 mm); Black = fishing line fragments (20 mm, caliber 0.30).

Overall, recovery efficiency of microplastic fragments was the same across all sizes and colours (origins), and only seems to be affected by time of stomach flushing, where lower recovery percentages were observed in the last days of our experiment (days 3 and 4). Size of microplastic particles evaluated in this experiment does not seem to affect transit time within the crocodiles’ digestive tract as large fragments (Size 1: between 2.36 and 4.75 mm) had similar recovery percentages to small fragments (size 2: between 2.36 and 1 mm) and the fishing line (20 mm, caliber 0.30). Highest recovery efficiencies were observed in the first two days after initial exposure (>80 %; Fig. 7).

**Fig. 8:**
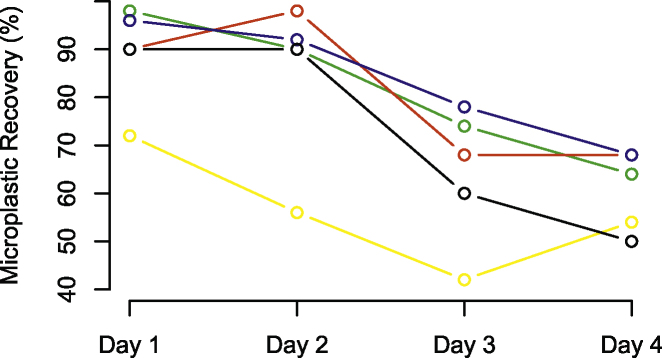
Recovery percentages of microplastic fragments by origin/colour. Blue, red and green represent fragments originating from polyethylene of ground soda bottle lid whilst yellow represent fragments originated from polyethylene low density of ground supermarket bag. Black line represents polyamide fragments of about 20 mm from a fishing line caliber 0.30.

The granular fragments of plastic (red, green and blue) and the fishing line exhibited the same pattern during the experiment, with recovery percentages greater than 80% within the first 48 h after exposure and a significant decrease in effectiveness in the following days. Yellow fragments, originating from Polyethylene low density bags, exhibited a recovery pattern that also tended to decrease with time, but recovery percentages of this fragment type was always inferior to 80 % (Fig. 8). We assume this to be a result of this fragment type having an elongated shape instead of granular, which can more easily be broken down into smaller pieces through the action of gastroliths present inside the crocodile’s stomach (fragments become then more likely to easily go through the digestive tract, which in turn affects the efficiency of recovery during stomach flushing).

These results further indicate the suitability of this method in recovery of microplastic fragments (polyethylene <4.75 to ≥1.0 mm) ingested by crocodiles, especially during the first two days after consumption.

## Declaration of Competing Interest

The authors declare that they have no conflict of interest.

## Appendix A Supplementary data

Dataset: Temperature (Celssius degrees) of water in the tanks with crocodiles
